# Serotonergic Contribution to Boys' Behavioral Regulation

**DOI:** 10.1371/journal.pone.0020304

**Published:** 2011-06-01

**Authors:** Amélie Nantel-Vivier, Robert O. Pihl, Simon N. Young, Sophie Parent, Stacey Ageranioti Bélanger, Rachel Sutton, Marie-Eve Dubois, Richard E. Tremblay, Jean R. Séguin

**Affiliations:** 1 Psychology Department, McGill University, Montreal, Canada; 2 Unité 669, Institut National de la Santé et de la Recherche Médicale (INSERM), Paris, France; 3 Université Paris-Sud and Université Paris Descartes, UMR-S0669, Paris, France; 4 Psychiatry Department, McGill University, Montreal, Canada; 5 School of Psychoeducation, University of Montreal, Montreal, Canada; 6 Department of Pediatrics, Centre Hospitalier Universitaire (CHU) Sainte-Justine, Montreal, Canada; 7 Psychology Department, Centre for Research on Human Development (CRDH), Concordia University, Montreal, Canada; 8 Departments of Psychology and Pediatrics, University of Montreal, Montreal, Canada; 9 School of Public Health, Physiotherapy and Population Sciences, University College Dublin, Dublin, Ireland; 10 Centre Hospitalier Universitaire (CHU) Sainte-Justine Research Centre, Montreal, Canada; 11 Department of Psychiatry, University of Montreal, Montreal, Canada; The University of Queensland, Australia

## Abstract

**Objectives:**

Animal and human adult studies reveal a contribution of serotonin to behavior regulation. Whether these findings apply to children is unclear. The present study investigated serotonergic functioning in boys with a history of behavior regulation difficulties through a double-blind, acute tryptophan supplementation procedure.

**Method:**

Participants were 23 boys (age 10 years) with a history of elevated physical aggression, recruited from a community sample. Eleven were given a chocolate milkshake supplemented with 500mg tryptophan, and 12 received a chocolate milkshake without tryptophan. Boys engaged in a competitive reaction time game against a fictitious opponent, which assessed response to provocation, impulsivity, perspective taking, and sharing. Impulsivity was further assessed through a Go/No-Go paradigm. A computerized emotion recognition task and a staged instrumental help incident were also administered.

**Results:**

Boys, regardless of group, responded similarly to high provocation by the fictitious opponent. However, boys in the tryptophan group adjusted their level of responding optimally as a function of the level of provocation, whereas boys in the control group significantly decreased their level of responding towards the end of the competition. Boys in the tryptophan group tended to show greater perspective taking, tended to better distinguish facial expressions of fear and happiness, and tended to provide greater instrumental help to the experimenter.

**Conclusions:**

The present study provides initial evidence for the feasibility of acute tryptophan supplementation in children and some effect of tryptophan supplementation on children's behaviors. Further studies are warranted to explore the potential impact of increased serotonergic functioning on boys' dominant and affiliative behaviors.

## Introduction

Investigations of serotonergic functioning reveal its complex contribution to various aspects of affective and behavioral regulation [1]. Studies focusing on serotonin's role in the regulation of social interactions and behaviors have paid special attention to 3 specific domains: hostility and aggression, dominance, and affiliation [2], [3].

Negative correlations between serotonergic functioning and aggression have been observed in both nonhuman primates and humans adults [2]. In monkeys, low cerebrospinal fluid (CSF) levels of the 5-HIAA metabolite are associated with high unrestrained violence, as well as impulsivity, risk taking behaviors, and premature violent death [4]–[8]. In humans, low serotonergic functioning has been associated with antisociality, impulsivity, and hostile aggression towards the self and others [9]–[12]. Manipulation studies have shown that decreased serotonin levels can increase hostility and aggression, while increased serotonergic functioning can decrease hostility and aggression [13]–[21].

Different patterns of association have emerged between serotonin and dominant behaviors which, unlike hostile aggression, are generally well-regulated, goal-oriented, and contribute to increase or maintain one's status. While variations were observed across species [22], [23], animal studies revealed positive associations between serotonergic functioning, dominance [24], [25], and affiliative behaviors [26]–[28]. Human studies replicated these findings [14], [20], [29], [30], and additionally found positive associations between serotonin and emotion recognition [31]–[34].

Developmental research has provided strong evidence for the early emergence of aggressive and prosocial behavioral tendency [35]–[38] as well as the predictive power of such tendencies for later adjustment [39]–[46]. However, the contribution of serotonin to children's behavioral regulation, as well as to the affective and sociocognitive mechanisms (e.g., emotional arousal, emotion recognition, perspective taking) underlying social interactions, is largely unknown. As described by van Goozen and colleagues [47], certain child studies have replicated adult findings of low serotonergic functioning and aggression (e.g. [48], [49]). However, positive correlations between serotonergic functioning and aggression(e.g., [50], [51]), or the absence of associations (e.g., [52], [53]) have also been reported in child samples [47]. Further, a positive association between serotonin levels and children's social competence has been reported [54].

Child studies have generally employed measures of central CSF metabolites (e.g., [48], [50]), fenfluramine challenge (e.g., [51], [52]) or peripheral indices of serotonergic functioning (e.g., [49]). In contrast, tryptophan is a dietary component and serotonin precursor which can be acutely augmented or depleted, respectively increasing and decreasing serotonin levels and thereby providing a highly useful window into serotonergic functioning [3]. Very few tryptophan manipulation studies have focused on child samples. Those that did administered tryptophan over a week or more to children with hyperactivity and inattention symptoms, yielding mixed findings [55]–[57]. A recent series of investigations however demonstrated that acute tryptophan depletion can increase children's response to provocation [58] and decrease their reaction times [59] during laboratory tasks. Tryptophan depletion also decreased behavioral inhibition in hostile children, while increasing behavioral inhibition in non-hostile children [60]. Never, to the best of our knowledge, has acute tryptophan supplementation been employed to investigate serotonergic contributions to children's behaviors.

Aggressive children are research participants of particular interest, as they often present deficits of behavioral and affective regulation, as well as problems within the sociocognitive processes underlying social interactions [44], [61], [62]. The present study thus investigated serotonergic functioning in physically aggressive boys, using a double-blind, acute tryptophan supplementation procedure. The purpose was to assess the feasibility of studying the behavioral effects of tryptophan in young children and to obtain a preliminary assessment of the effect of tryptophan. We focused on boys' response to provocation, impulsivity, affiliative behaviors, perspective taking, and emotion recognition. In light of previous findings of increased serotonergic functioning leading to low aggression and high dominance, we hypothesized that boys in the tryptophan group would be dominant but non-aggressive when responding to provocation by a fictitious opponent. Specifically, we expected boys in the tryptophan group to better adjust their responses as a function of the level of provocation than boys in the control group. Furthermore, we expected boys in the tryptophan group to show lesser impulsivity than boys in the control group. Finally we hypothesized that boys in the tryptophan group would show greater sharing and helping behaviors, as well as greater perspective taking and emotion recognition, than boys in the control group.

## Methods

### Participants

Participants for the present study were boys from a community sample of 572 children who have been followed yearly since they were 5 months old [63]. Our sample was determined by the availability of boys for whom a longitudinal assessment of behaviors indicative of a high probability of long term elevated physical aggression was available. Specifically, maternal ratings of child behaviors within the past year were obtained on six occasions when the children were between 17 and 84 months of age, using items from the Preschool Behavior Questionnaire [64]. Mothers rated the frequency (0-never, 1-sometimes, 2-often) of the following behaviors: 1) Got into fights; 2) Physically attacked people; 3) Hit, bit, or kicked other children.

**Figure 1:**
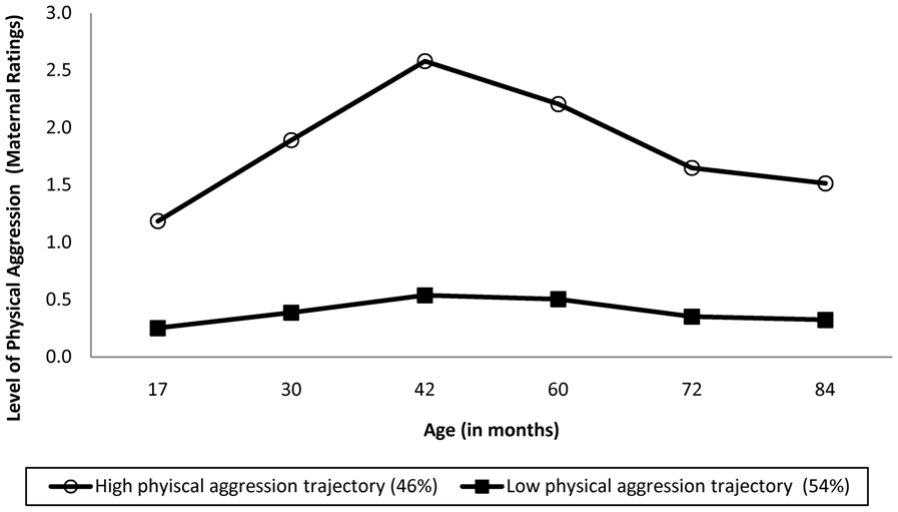
Physical aggression trajectories from 17 to 84 months.

Developmental trajectories of physical aggression [65], [66], spanning ages 17 to 84 months, were modeled based on mother ratings for 512 children of the original sample.The developmental trajectory method allows for a summary of population heterogeneity on a certain characteristic, over time. Polynomials are used to represent developmental trajectories varying in level and shape, identifying subgroups of individuals displaying distinct patterns of behaviors over time [66]. Group membership in trajectory models is not absolute, as represented by the posterior probabilities of group membership, consisting in the probability for each individual of belonging to each trajectory estimated from the sample. The maximum probability rule is used to assign individuals to the trajectory to which they have the highest probability of belonging (see Nagin [66] for a complete discussion of developmental trajectory analyses). As shown in Figure 1, a two-group model of physical aggression over time was estimated in which 46 percent of children followed a high developmental trajectory whereas 54 percent of children followed a low/stable developmental trajectory. Within the high physical aggression trajectory 103 boys had a posterior probability in the top quartile. Families of 59 of these boys could be contacted. Exclusion criteria were the presence of a serious medical illness, history of head injury, and current use of prescription medications, with the exception of stimulant medication. Participants taking one of the exception drugs had to be on the same dosage regimen for at least one month to be included in the study. They were also asked not to take their medication on the day of testing, in line with the indicated medication washout period. One boy was excluded from participation based on the medication criteria. Two boys were excluded due to the presence of a medical illness (one with a diagnosis of diabetes, one with a diagnosis of epilepsy). The parents of 23 of the 56 remaining boys consented to participate. Mean age at the time of the study was 123.2 months (SD = 2.8 months).

Of the 33 families who refused participation, 15 parents stated they did not have time to participate or did not want to travel to the laboratory, five were uncomfortable with their son taking tryptophan, three did not give reasons specific to the experiment but wanted to withdraw from the larger longitudinal research program, and one did not want their son to skip a day of their stimulant medication. Refusal came from the boys themselves in four additional cases. Reason for refusal is unknown for the remaining five families. In order to assess whether participating children significantly differed from non-participating children, we compared the 23 participating boys with the remainder of the initial 103 identified boys on mother and family characteristics (i.e., family structure, maternal education, maternal age at birth of the first child, maternal depression, maternal antisocial tendencies) at entry within the longitudinal program and boys' probability of belonging to the high physical aggression trajectory. No significant differences were noted. Participants in the present study may thus be considered representative of the initially targeted sample.

The research protocol was sanctioned by Health Canada (No-objection letter reference 9427-S1805-33C), the Ste-Justine Hospital Research Center Ethics Committee, and the McGill University Institutional Review Board (IRB) of the Faculty of Medicine. Informed verbal and written consent were obtained from parents of all our participants, while informed verbal and written assent to participate were obtained from all participants. Participants were treated according to the American Psychological Association principles [67].

### Procedure

Informed parental verbal and written consent and boys' assent were obtained upon arrival at the laboratory. The present study followed a randomized, double-blind design. Group assignment for the first participant was randomly determined by flipping a coin. A sequence alternating between each condition followed for subsequent participants. This randomization method was used in light of the small sample size and the absence of clear cut-off points for stratification of randomization within physical aggression probability ranges, our selection measure. All boys were given a chocolate milkshake which, for 11 boys (the tryptophan group), contained a 500 mg tryptophan (Tryptan^TM^) tablet previously ground into a fine powder. The remaining 12 participants (the control group) received a chocolate milkshake without tryptophan. Only the onsite research supervisor, who did not interact with families and participants during the experimental procedures, knew of the group assignment. Therefore, the research assistants interacting with participants and their parents, as well as parents and the boys themselves were blind to group assignment and the randomization procedure used. Comparison of the two groups through independent sample *t* tests on age at the time of the study and background behavioral and sociodemographic characteristics revealed no significant differences. The groups may therefore be considered equivalent.

Experimental tasks were administered 45 minutes following milkshake ingestion. This was done in order for enough serotonin to be synthesized from tryptophan to have an effect on brain function [68]. The sequence of task presentation was the same for all participants. The testing session was filmed in its entirety to allow subsequent coding.

### Measures

#### Competitive reaction-time game

A modified version of a competitive reaction-time game elaborated by Pelham and colleagues [69] was administered. Boys were informed they would play a game with another boy over the Internet, when in fact the opponent was fictitious and boys were playing against the computer. A video of the fictitious opponent “getting ready to play”, a child confederate previously filmed, was presented to participants before the game began.

The game consisted of pressing the space bar and releasing it as quickly as possible when the computer gave a signal. Boys were instructed that the player with the faster reaction time on any given trial would be awarded 50 points. Additionally, the winner could remove 0 to 100 points from their opponent. These points would not go to the player taking points away, but to a common point “bank”. This was done so that removing points from the opponent would not provide instrumental gains. Participants were told that the competitor who won the most trials would be allowed to trade his points for a toy at the end of the game.

The sequence of wins and losses, as well as the number of points the computer took away from the boys on each loss trial, was predetermined. There were a total of 40 trials, divided into quarters of ten trials, varying in provocation level. There were no pauses between the quarters. The first was a “no provocation” baseline, where the computer did not remove points from participants when they lost. The second consisted of a “high provocation” period, where the computer removed 80 or 90 points when boys lost. The third was a “low provocation” period, where the computer removed 10 or 20 points when boys lost. Finally, the last quarter was again a “no provocation” period, where the computer did not remove any points when boys lost. Final earnings for all participants were 900 points.

Boys' response to provocation was operationalized as the number of points they took away from their opponent, as well as the time they took in deciding how many points to remove, at each quarter relative to the no provocation baseline. Impulsivity was indexed by the number of times the boys released the spacebar before the signal was given by the computer. Reaction times were used to assess whether both groups were equally vigilant and engaged throughout the task.

We extended Pelham et al.' s [69] original protocol to include measures of perspective taking and sharing. Boys indicated how they thought their opponent was feeling upon losing the game, using an array of schematic faces presented on the computer (neutral, happy, sad or angry). Sharing behaviors were assessed next. The research assistant showed the boys a video of the fictitious opponent looking sad and disappointed for having lost. Boys were told that, as the winner, they could divide the points from the bank as they wished between themselves and their opponent. The experimenter emphasized that the decision would be anonymous. Boys were left alone to divide the points, using an analog scale on the computer.

#### Go/No-Go

The basic Go/No-Go paradigm measures inhibitory control [70]. Participants were instructed that the letter ‘X’ or ‘Y’ would appear on the screen, and that they should press the space bar only when a ‘Y’ appeared, and not press any key when an ‘X’ appeared. One hundred trials were administered. Each stimulus was on the screen for 300 ms and the intertrial period lasted 700 ms. The number of successful trials, the number of omission errors, and the number of commission errors were recorded.

#### Ring incident

A staged incident was implemented to measure boys' helpful behaviors. As the boys were getting ready to take part in a computer activity, the experimenter looked at her hand, gasped and said: “Oh no! I lost my ring!” The experimenter then searched the room according to a set routine, allowing boys time to help. The incident ended with the experimenter finding the ring, previously hidden in the testing room. Videos of the incident for all 23 children were first coded by a trained rater, to verify whether (yes or no) children 1) noticed the incident, 2) verbally expressed concern, 3) visually scanned the room to search for the ring, or 4) physically got up from their chair to help the experimenter find the ring. A second trained rater coded a random selection of 30% of the videos for reliability. Mean interrater agreement ranged from 71% to 100% (kappa ranging from 0.30 to 1.00).

#### Emotion Recognition

An emotion recognition task developed by Pollak and Kistler [71] was administered. Participants were asked to identify the emotion displayed in pictures of adults making different facial expressions. The pictures consisted of blended images of the models displaying four basic emotions: happiness, sadness, fear and anger. Thus, the pictures represented a continuum between two emotions, for example happy-fearful or angry-sad, in increments of 10%. Participants were asked to identify the emotion shown in 224 trials including both the prototypic and blended pictures, always choosing between two response options. All trials began with a central fixation point for 250 ms and ended with a blank screen for another 250 ms. Mean accuracy (%) of identification for each continuum was the main dependent measure.

### Analyses

For the competitive reaction-time game, group differences in number of points taken away from the “opponent”, decision time, reaction time, percentage of points shared, and impulsivity (i.e., number of inappropriate spacebar releases) were investigated with *t* tests, whereas group differences in perspective taking were assessed through Fisher's exact test. Within-subject repeated measures analyses of variance were performed separately for each group to assess patterns of responses to varying levels of provocation. The number of points taken away and decision time during the high provocation (phase 2), low provocation (phase 3) and no provocation (phase 4) phases were standardized (z scores) using the no provocation baseline (phase 1) means. These standardized values were then used in planned comparisons to assess the extent of deviation from baseline responding. Group differences were assessed using *t* tests for the Go/No-Go and emotion recognition tasks. Finally, Fisher's exact test was used to evaluate group differences on help behaviors during the staged ring incident. Analyses were completed using SPSS 15 [72] and R 2.10.1 software [73].

## Results

### Response to the competitive reaction time game

Table 1 presents results of independent sample *t* tests investigating group differences in response to provocation during the competitive reaction time game, specifically overall number of points taken away, overall mean decision time, and overall mean reaction time. No significant group differences emerged. However, the boys in the tryptophan group tended to be less impulsive than controls, as measured by the number of inappropriate spacebar releases made (*t*(21)  = 1.85, p = 0.08).

**Table 1:** Group differences on experimental tasks.

	ControlMean(SD)	TryptophanMean (SD)	*t* (df)	*p*	*Cohen d*
Competitive reaction time game					
Total points taken away	1040.00 (525.13)	1347.27 (730.88)	-1.17 (21)	0.26	0.49
Overall mean decision time	1.53 (0.51)	1.35 (0.40)	0.96 (21)	0.35	-0.39
Overall mean reaction time	0.33 (0.03)	0.33 (0.05)	-0.06 (21)	0.95	0.00
Impulsivity	2.92 (1.88)	1.64 (1.36)	1.85 (21)	0.08	-0.77
% of bank points shared	0.70 (0.23)	0.66 (0.30)	0.29 (21)	0.77	-0.15
Go/No-Go					
Number of successes	61.25 (14.76)	58.09 (16.53)	0.48 (21)	0.63	-0.20
Number of omissions	34.08 (16.03)	37.91 (17.07)	-0.55 (21)	0.59	0.23
Number of commissions	4.67 (3.14)	4.00 (2.41)	0.57 (21)	0.58	-0.24
Emotion recognition					
Happy-Sad accuracy	0.84 (0.06)	0.84 (0.05)	0.24 (21)	0.82	0.00
Happy-Fear accuracy	0.85(0.08)	0.91 (0.08)	-1.82 (21)	0.08	0.75
Mad-Sad accuracy	0.88 (0.07)	0.88 (0.05)	-0.07 (21)	0.94	0.00
Mad-Fear accuracy	0.82 (0.10)	0.85 (0.10)	-0.68 (21)	0.51	0.30

**Figure 2:**
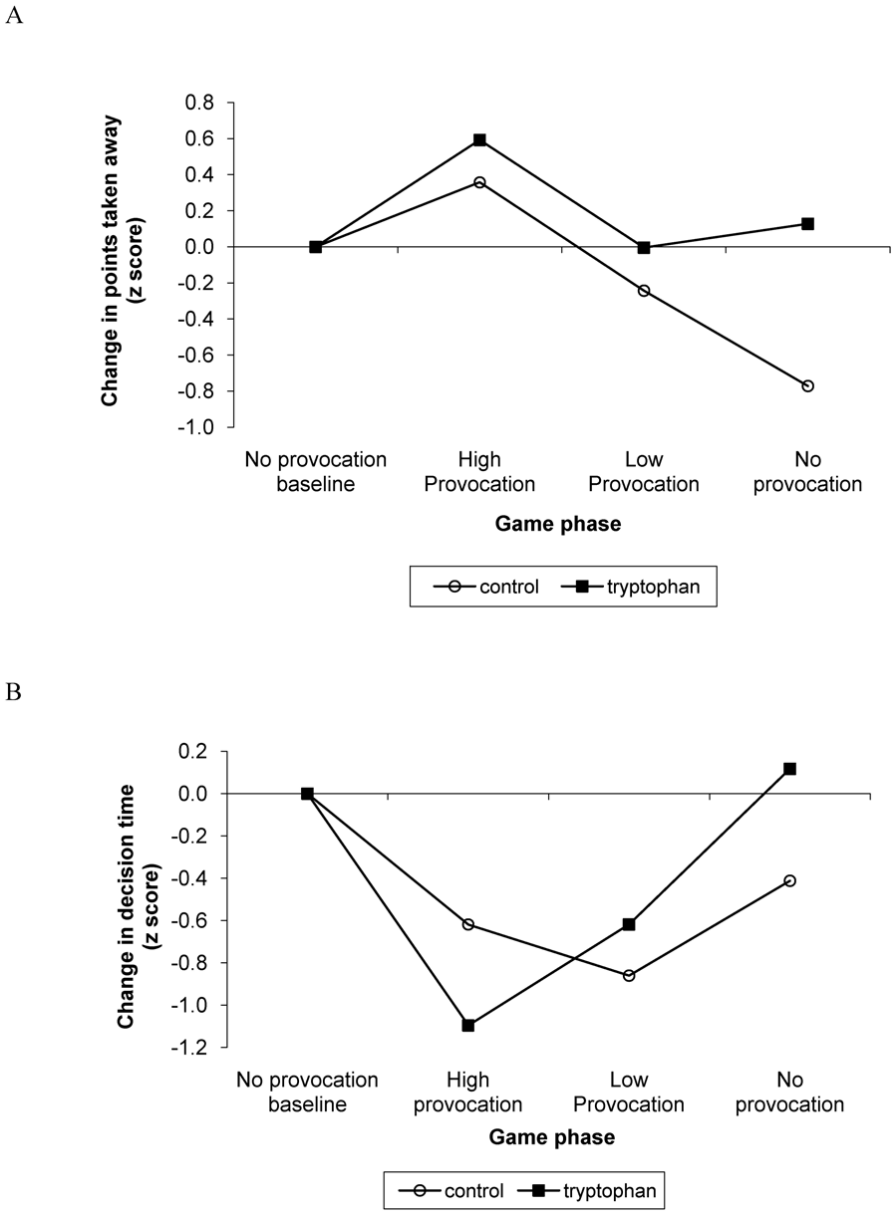
Points taken away (A) and decision time (B) at each phase of the competitive reaction time game. Values represent change (z score) in points taken away and decision time at each phase of the game, relative to the no provocation baseline.

Within-subject repeated measures analyses were performed separately for each group to assess patterns of response to varying levels of provocation by the fictitious opponent. The groups did not differ at baseline on points taken away and decision time. As described above, scores on these measures were standardized relative to the no-provocation baseline. As illustrated in Figure 2a, provocation level had a significant effect on points taken away for the control group (*F*(1.46)  = 7.37, *p* = 0.01) but to a lesser extent for the tryptophan group (*F*(3)  = 2.30, *p* = 0.10). Planned comparisons revealed that boys in the control group tended to take away more points than at baseline during the high provocation phase (*z* = 0.36, *F*(1)  = 3.81, *p* = 0.08, *d* = 0.45) and significantly decreased the number of points they took away during the last phase of the game (*z*  =  −0.77, *F*(1)  = 5.00, *p* = 0.05, *d*  =  −0.93). For the tryptophan group, the only significant change in number of points taken away relative to baseline was an increase during the high provocation phase of the game (*z* = 0.59, *F*(1)  = 6.31, *p* = 0.03, *d* = 0.64). Groups differed in points taken away during the last phase (*t*(15.43)  =  −2.40, p = 0.03). As shown in Figure 2b, provocation level had a significant effect on decision time for both the control group (*F*(3)  = 4.06, *p* = 0.02) and the tryptophan group (*F*(1.84)  = 4.33, *p* = 0.03). Boys in the control group significantly decreased their decision time relative to baseline during the high provocation (*z*  =  −0.62, *F*(1)  = 4.87, *p* = 0.05, *d*  =  −0.72) and low provocation (*z*  =  −0.86, *F*(1)  = 10.21, *p* = 0.01, *d*  =  −0.95) phases of the game. Boys in the tryptophan group significantly decreased their decision time relative to baseline only during the high provocation phase (*z*  =  −1.10, *F*(1)  = 16.36, *p*<0.01, *d*  =  −1.26).

**Figure 3:**
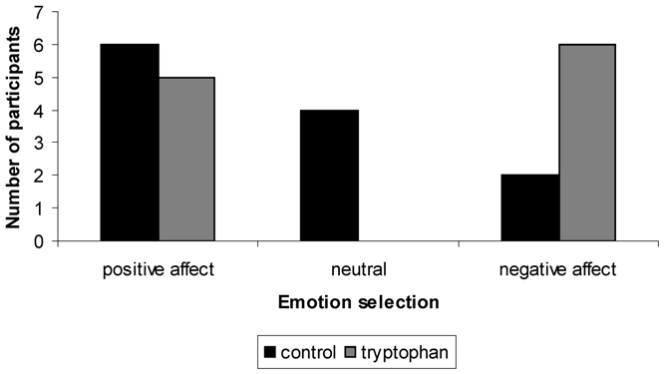
Perspective taking following the competitive reaction time game. Fisher's exact test (p = 0.07).

As shown in Table 1, no significant group differences emerged for the percentage of points shared with the fictitious opponent at the end of the game. However, Figure 3 shows a trend for boys in the tryptophan group to be less likely to describe their defeated opponent as emotionally neutral, and instead tend to describe the opponent as experiencing a negative emotional state (Fisher's exact test, p = 0.07).

### Go/No-Go

As shown in Table 1, no group differences emerged on number of successful trials, number of omission errors, and number of commission errors.

### Emotion recognition

A statistical trend for the tryptophan group to be more accurate in distinguishing happiness and fear (*t*(21)  =  −1.82, *p* = 0.08) was observed. This trend represents a large effect size (*d* = 0.75). No significant group differences emerged on the other continua.

### Reaction to the lost ring incident

**Figure 4:**
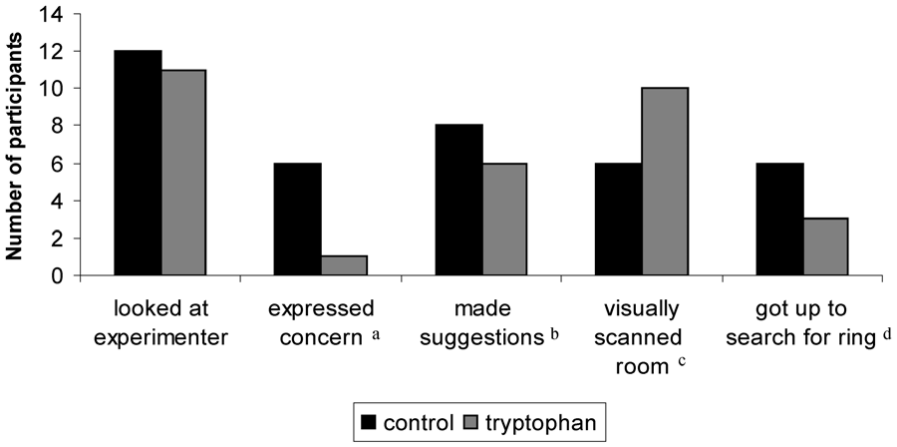
Reaction to the lost ring incident. ^a^ Fisher's exact test (p = 0.07). ^b^ Fisher's exact test (p = 0.68). ^c^ Fisher's exact test (p = 0.07). ^d^ Fisher's exact test (p = 0.40).

As shown in Figure 4, more children in the control group tended to verbally expressed concern regarding the lost ring (Fisher's exact test, *p* = 0.07), while children in the tryptophan group tended to be more likely to visually scan the room in search of the ring (Fisher's exact test, *p* = 0.07). No further trends were noted.

## Discussion

The present study investigated serotonergic functioning in boys with a history of behavior regulation difficulties, using a double-blind acute tryptophan supplementation procedure. It was hypothesized that tryptophan supplementation could lead to a more dominant and less aggressive pattern of responding to provocation, while decreasing impulsivity and increasing perspective taking, sharing, helping, and emotion recognition. These hypotheses were partially supported.

When competing against a fictitious opponent, boys in both the tryptophan and control groups increased the number of points they took away during the high provocation phase (phase 2) relative to baseline. However, while boys in the tryptophan group returned and stayed at baseline level for the remainder of the game, boys in the control condition significantly decreased the number of points they took away from their opponent during the last phase. Boys in the tryptophan group also tended to be less impulsive than boys in the control group when competing against the fictitious opponent, making fewer inappropriate spacebar releases. Findings of decreased response to provocation by the control group are not consistent with past studies of acute tryptophan manipulation and laboratory aggression [17], [18], [58]. Such patterns may however be interpreted in light of studies investigating the impact of increased serotonergic functioning on non-human primates and human adults' dominant behaviors [26], [29]. From this perspective, boys in the tryptophan group may have been better able to sustain the competitiveness of the reaction time game, remaining focused and maintaining their level of responding unless highly provoked. In contrast, boys in the control group appeared to have been less able to sustain the competitiveness of the game, decreasing the amount of points they took away towards the end of the game. It is important to note however that, while a trend was observed for impulsivity during the competitive reaction time game, no group differences emerged during the Go/No-Go task. It is therefore possible that the tendency for lesser impulsivity in tryptophan boys may be specific to socially competitive and engaging contexts.

Furthermore, children in the tryptophan group tended not to select neutral schematic faces but rather to select negative schematic faces when describing their defeated opponent. They similarly tended to be more accurate than controls in distinguishing between facial expressions of happiness and fear. They tended to be more proactive and helpful, visually scanning the laboratory room for a ring the experimenter pretended to lose. Trends of greater perspective taking and helpful behaviors in the tryptophan group may be viewed as consistent with the literature on increased serotonergic functioning and affiliative behaviors in non-human primates and human adults. However, we must also emphasize that children in the control group tended to be more likely to express verbal concern than children in the tryptophan group in response to the lost ring incident.

To the best of our knowledge, the present study was the first to implement an acute tryptophan supplementation procedure with children. Our results, as well as the conclusions that may be drawn from them, are therefore preliminary. Specifically, the group sizes were small, limiting our power to detect statistically significant differences between the groups. A number of marginally significant findings (p<0.10) emerged, particularly with regards to affiliative and prosocial responding, and were treated as trends. As reviewed by Abelson [74], findings with p values of this magnitude have been described as leaning towards significance or providing hints of significance, and would be potentially relevant. We would like to suggest that the trends observed in the present study do not allow us to draw firm conclusions regarding associations of serotonergic functioning and behavior regulation in children, but constitute interesting avenues for future studies that should be verified through replication within larger samples.

However, while replication is necessary, the pattern of results observed within the present study does fall partially in line with some of the previous literature on serotonergic functioning and social behaviors. We may tentatively speculate that boys in the tryptophan group tended to show more dominance, helpfulness, and affiliative responding. Such a pattern of response could be underlined by increased emotion regulation, leading to more situationally appropriate and goal-directed behaviors. In contrast, the decreased response to provocation and concern expressed by boys in the control group could stem from greater difficulties in emotion regulation, which may interfere with goal-directed behaviors appropriate to the context at hand. Future studies specifically investigating the impact of acute tryptophan supplementation on affective arousal and regulation are needed to verify this potential mechanism.

In addition, future studies of acute tryptophan supplementation in children may benefit from the use of various methodologies. The laboratory context of the present investigation allowed for a standardization of the stimuli and conditions presented to the children, as well as a rigorous comparison of the two groups. The extent to which results obtained in laboratory settings may generalize to more naturalistic settings is however an important question. Extending the acute tryptophan supplementation methodology in children to various and more naturalistic contexts will be highly important. Also, the competitive reaction time game used in the present study follows from a long tradition of laboratory tasks inspired by the original Taylor-Buss paradigm [75], where participants were typically given the option of administering electrical shock to their fictitious opponent. However, it may be argued that, unlike the version of the task used in the present study, the original paradigm used by Taylor was more likely to elicit aggression rather than dominance. It will be necessary to investigate the effect of tryptophan supplementation on children's response to provocation using tasks tapping a greater range of social behaviors, including more hostile forms of responding [76], as well as different sequences and patterns of provocation, to disentangle the meaning of the responses observed. Also important for clarification would be the use of competitive games where the outcome is maximized for participants through cooperation with their opponent. For example, acute tryptophan depletion decreased levels of cooperation in healthy adults during a prisoner's dilemma paradigm [77]. Findings of greater cooperation on such a task from children having received tryptophan would support the hypothesis that tryptophan increases emotion regulation, as well as adaptive, and situationally appropriate responding. Finally, the majority of previous studies have focused on acute tryptophan depletion and aggression. Whether acute tryptophan depletion and supplementation are equally powerful in influencing behaviors, particulary in younger samples, should be verified.

In sum, the present study provides preliminary evidence for the feasibility of acute tryptophan supplementation in children and some effect of tryptophan supplementation on children's behaviors. Future studies attempting replication and expanding on the present methodology will be helpful in clarifying the nature and meaning of relationships between serotonin, aggression, dominance, and affiliation in children.
